# Aging and the rise of somatic cancer-associated mutations in normal tissues

**DOI:** 10.1371/journal.pgen.1007108

**Published:** 2018-01-04

**Authors:** Rosa Ana Risques, Scott R. Kennedy

**Affiliations:** Department of Pathology, University of Washington, Seattle, Washington, United States of America; Case Western Reserve University School of Medicine, UNITED STATES

## Abstract

DNA mutations are inevitable. Despite proficient DNA repair mechanisms, somatic cells accumulate mutations during development and aging, generating cells with different genotypes within the same individual, a phenomenon known as somatic mosaicism. While the existence of somatic mosaicism has long been recognized, in the last five years, advances in sequencing have provided unprecedented resolution to characterize the extent and nature of somatic genetic variation. Collectively, these new studies are revealing a previously uncharacterized aging phenotype: the accumulation of clones with cancer driver mutations. Here, we summarize the most recent findings, which converge in the novel notion that cancer-associated mutations are prevalent in normal tissue and accumulate with aging.

## Introduction

DNA encodes the basic instructions to construct an organism during its development, and its stability is essential to life. However, DNA mutations are also necessary for evolution because they provide the requisite genetic variation for natural selection. Mutations are passed to the offspring via the parents’ germline, producing iterative cycles of mutagenesis and selection that allow organisms to adapt to changing environmental conditions. Thus, opposing forces act on DNA maintenance: stability to preserve the quality of the genetic information within individuals and instability to warrant intergenerational genetic diversity [1].

For new genetic information to have its phenotypic effect, the zygote must divide and clonally expand during embryonic development up to 10^13^ to 10^14^ cells in humans [2]. While the cells that make up the resulting organism may differ in morphology and physiology, their underlying genetic code should be, in principle, identical. However, much like how genetic variation drives selection within organismal populations, genetic variation arising in the soma enables selection for or against somatic cells. The stochastic nature of mutagenesis, the sparse gene content of the human genome, and the limited degeneracy of the genetic code imply that most mutations have neutral or deleterious consequences. Occasionally, however, mutations provide a selective advantage that leads to the expansion of the mutant cell into a clone. This process can be influenced by the timing of mutations during an organism’s lifecycle, their frequency, and their functional consequence to a cell’s physiology. The result is genetically distinct populations of cells within the soma of an individual, a phenomenon known as somatic mosaicism [3].

**Fig 1:**
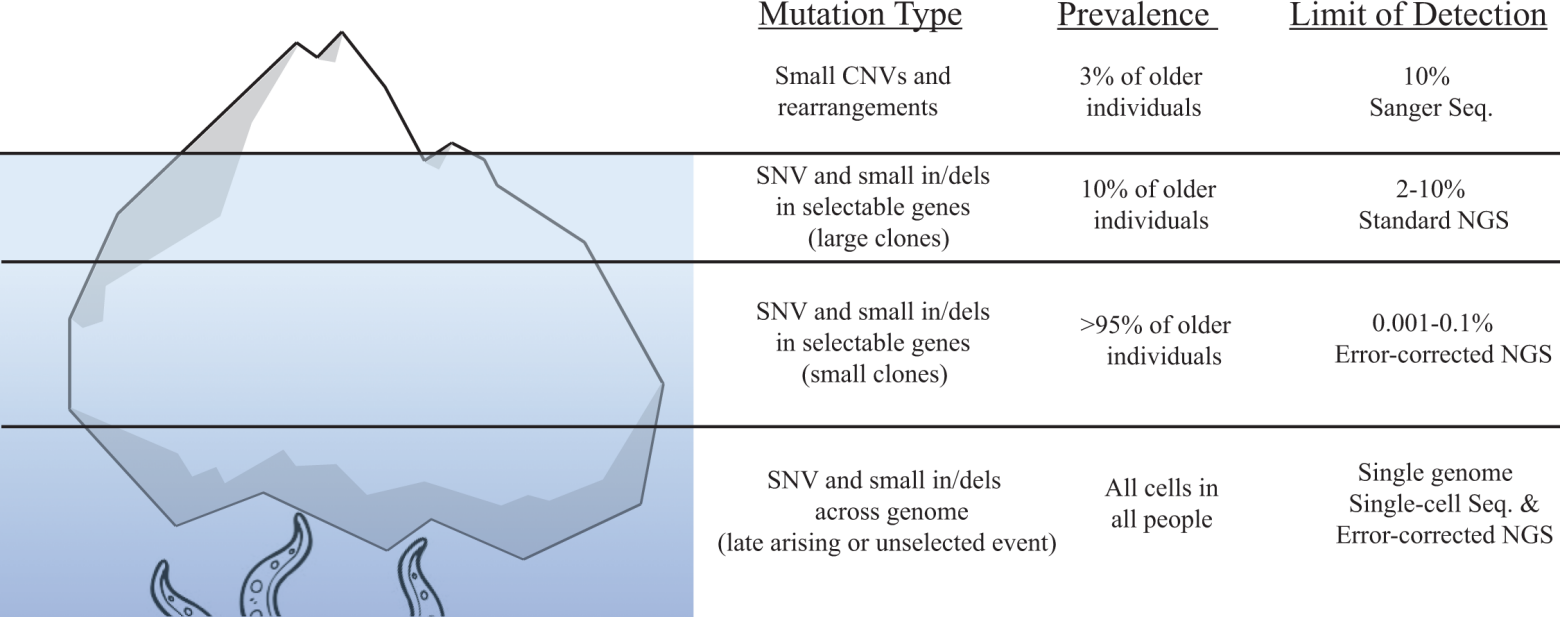
The hidden burden of somatic mutations. The extent of somatic mutations in healthy tissues can be thought of as an iceberg, such that the true prevalence of these mutations is only now being recognized as technologies have improved (right column). The limit of detection refers to the ability to identify a certain mutation within a given biopsy. The cumulative results of recent studies have shown that cancer-associated mutations (left column) are found in the population with a prevalence (middle column) that is indirectly proportional to the size of the clones and the age of the individuals. That is, large clones (>10% MAF of a given biopsy) have low prevalence and are typically found only in old individuals, whereas small clones (<0.1%) are very prevalent, also at mid age. CNV, Copy Number Variant; ddPCR, Digital Droplet PCR; in/dels, insertions and deletions; iPSC, Induced Pluripotent Stem-Cell; MAF, Mutant Allele Fraction; NGS, Next Generation Sequencing; RT-PCR, Real Time Polymerase Chain Reaction; SNP, Single Nucleotide Polymorphism; SNV, Single Nucleotide Variant.

The existence of somatic mosaicism is well documented. While outside the scope of this review, a number of rare diseases are attributed to mosaicism that arises during the first few divisions of an embryo, with the severity and phenotypic expression being influenced by when in development they occur [4–6]. However, the occurrence of somatic mosaicism is not limited to development and has been recognized as an aging phenotype for decades (Reviewed in [7]). An increase of somatic mutations with age has been reported for a variety of target genes, including HLA-A [8], hypoxanthine phosphoribosyl transferase (HPRT) [9–13], T-cell receptor [14], and glycophorin A [14]. Similarly, age-associated accumulation of chromosomal alterations has been documented with a variety of cytogenetic approaches, from chromosome painting [15] to single nucleotide polymorphism (SNP) arrays [16–18]. These early findings appear to be only the tip of an iceberg in terms of somatic mutations in normal tissue (Fig 1). The advent of Next Generation Sequencing (NGS) technologies has increased the resolution of mutation detection down to approximately 1% for single base substitutions and has led to the striking revelation that older individuals not only accumulate chromosomal alterations but also abundant mutations in cancer driver genes [19–22]. These initial NGS studies in blood reported cancer-associated mutations in approximately 10% of individuals older than 65. However, as error-correction NGS (ecNGS) technologies have improved the limit for mutation detection, the prevalence of cancer-associated mutations in adults appears closer to 100% [23,24]. Furthermore, recent single-cell studies point to the possibility that essentially all cells have unshared mutations in their genomes [25–27]. In view of this extensive genetic diversity, it is perhaps not surprising that mutations that confer a proliferative advantage are readily detected as clonal populations of increasing abundance and size in the elderly. These clonal populations might lead to loss of organismal health through the functional decline of tissue and/or the promotion of disease processes, such as cancer. In this review, we summarize recent research that supports the notion that aberrant clonal expansion (ACE; originally formulated in Forsberg et al. [28]) resulting from cancer-associated mutations are common in noncancerous tissue and accumulate with age. We propose ACE to be a previously underappreciated aging phenotype that is universal in most organisms, affects multiple tissues, and likely helps explain why aging is the biggest risk factor for cancer.

## Somatic mutations in blood

Due to its proliferative nature and ease of sampling, the hematopoietic compartment is where somatic mutations have been most frequently studied. Variants include both large structural chromosomal alterations and point mutations affecting cancer-associated genes. The first reports in the mid-1990’s indicated that up to one-third of normal adults harbored gene fusion events commonly found in leukemias and lymphomas in their blood. These mutations include the *BCR-ABL* fusion event, associated with chronic myeloid leukemia (CML) and acute lymphoblastic leukemia (ALL) [29,30], and the *BCL2-IGH* and *IGH-cMyc* fusion events, associated with follicular non-Hodgkin lymphoma [31,32]. Interestingly, some translocations appear to occur very early in life. For instance, a study of cord blood found the leukemia-associated *TEL-AML1* and *AML1-ETO* gene fusion events are present in approximately 1% of neonates [33].

More recently, the combined analysis of several genome-wide association studies (GWAS) collectively comprising more than 100,000 individuals indicated the presence of large (>2 Mbp) leukemia-associated chromosomal abnormalities, such as aneuploidy and copy-neutral loss of heterozygosity, present in the peripheral blood and buccal swabs from patients free of clinically detected malignancies [16,17]. Age was found to be the only significant predictor of mosaic status; the frequency of these events was low in the cohort younger than 50 years (<0.5%), but this frequency rapidly increased to 2% to 3% of individuals in their 70’s and 80’s [16,17]. As indicated in Fig 1, the limit of detection of SNP arrays, which was the technology used in these studies, is approximately 10% mutant allele fraction (MAF) within the sample. This led the authors to speculate that the true prevalence of these mutations at levels below their limit of detection was likely significantly higher in the general population [17]. Importantly, many of the reported chromosomal abnormalities overlapped with numerous genes known or suspected to be involved in hematological cancers. These include *DNMT3A* and *TET2*, commonly deleted in myelodysplastic syndrome, myeloproliferative disorder, and acute myeloid leukemia [34,35]; *PRAME*, *DLEU7*, *DLEU1*, and *DLEU2*, frequently deleted in chronic lymphocytic leukemia, the most common leukemia in older adults [36–39]; *L3MBTL1*, a putative tumor suppressor in myeloid disorders harboring del(20q12) deletions [40]; and *RB1*, a well-studied tumor suppressor mutated in many leukemias [41].

An important milestone in the field occurred in 2014 with the publication of three large population studies using whole exome sequencing. The studies reported abundant mutations in the peripheral blood of older healthy individuals [19–21]. The genes most frequently mutated were *DNMT3A*, *TET2*, *ASXL1*, and *JAK2*, which are genes implicated in myelogenous leukemias. This phenomenon was termed clonal hematopoiesis of indeterminate potential (CHIP), but it was later referred to simply as clonal hematopoiesis [42,43] or ACE. We favor ACE because it reflects the concept of abnormal expansion, and it is generally applicable to expansions in all somatic tissues. In the three original studies, ACE was reported to be infrequent in individuals less than 50 years of age but rapidly increased in prevalence to approximately 10% of individuals after age 65 and 18% after age 90 [19–21]. The presence of these clonal expansions conferred a small but significant risk of leukemia (0.5%–1% per year), suggesting that these clones represent an early stage of leukemic progression [20,44]. Recently, a second round of studies have taken advantage of the increased sensitivity of ecNGS technologies (Fig 1) to further characterize ACE in blood. Using ecNGS and digital droplet PCR, Young et al. identified mutations in *DNMT3A* and *TET2* in 95% of healthy 50- to 60-year-old individuals at frequencies ranging from 0.03% to 14% [23]. Using a different ecNGS modality, Acuna-Hidalgo et al. reported clonal hematopoiesis with driver mutations at all ages tested, ranging from 3% of individuals 20 to 29 years of age to 20% of individuals 60 to 69 years of age [45]. In a large cohort of more than 11,000 Icelanders, Zink et al. reported that ACE inevitability occurs in the elderly, and, regardless of identifiable driver mutations, clonal expansions were associated with risk of hematological malignancy [42].

Interestingly, the risk for a number of nonmalignant diseases correlates with the presence of mutations in cancer driver genes. For example, Jaiswal et al. noted in their original study that, in addition to an increased risk for hematological malignancy, the rate of all-cause mortality, coronary heart disease, and ischemic stroke was also increased [20]. Zink et al. also noted a similar association with mortality as well as psychiatric disease, smoking-related diseases, and chronic obstructive pulmonary disease (COPD) but not nonhematological malignancies [42]. Statistical correction for smoking status, which is known to correlate with these phenotypes, still resulted in a significant correlation. However, smoking status was not well documented in this cohort, leaving open the possibility that the statistical correction was confounded. An accompanying study also reported an age-dependent increase in *TET2* and *DMNT3A* and, interestingly, did not observe an increase with any comorbidities except COPD asthma [46]. Separately, ACE has also been associated with loss of chromosome Y, which is a well-characterized aging feature that correlates with shorter survival and increased nonhematological cancer risk [47].

The association between mutations in cancer driver genes and vascular disease is surprising. A follow-up study by Jaiswal and colleagues specifically designed to address this relationship has recently shown a significant correlation between somatic mutations in *DNMT3A*, *TET2*, *ASXL1*, and *JAK2* and atherosclerotic cardiovascular disease [48]. These findings are further supported in a *Tet2*-knockout mouse that exhibited significantly larger atherosclerotic lesions and elevated expression of chemokine and cytokine genes involved in atherosclerosis, potentially acting through an interleukin 6 (IL-6)–mediated increase in inflammation [48,49]. Taken together, these studies have revealed that blood ACEs are surprisingly prevalent with aging and that they are significantly associated with environmental exposures, a variety of age-related diseases, and mortality. Further research is needed to clarify these intriguing associations.

Evidence indicates that these mutations are not static and are likely under selective pressure. For example, a number of environmental factors, such as smoking, viral infections, and pesticide exposure, correlate with clonal expansion, suggesting that these environmental factors may contribute not only through mutation induction but also by modulation of clonal expansion [32,47, 50]. The fact that the MAF of clones increases yearly supports a continuous clonal expansion [46]. However, longitudinal follow-up of clones has revealed that they are mostly stable over time, suggesting that these mutations might not confer an increase in proliferation but an elevated self-renewal capacity [23]. In addition, not all clones seem to be positively selected or persist through life. Forsberg et al. observed a gain and subsequent clearance of a large clone containing copy number variant (CNV) in chromosome 4q in an individual over the course of 19 years [18]. Additionally, the frequency of leukemia-associated gene fusion events in newborns is approximately 100-fold more prevalent than the cumulative frequency of leukemia before the age of 15 years [33]. Thus, the role of selection in ACE remains unclear and would benefit from studies that include longitudinal sampling.

## Somatic mutations in solid tissues

Mutations in cancer driver genes have been reported in histologically normal tissues for decades but almost exclusively in the context of preneoplastic diseases, such as ulcerative colitis, Barrett’s esophagus, or normal tissue adjacent to tumors [51–54]. In those preneoplastic fields, somatic mutations are abundant and have been extensively analyzed, most notably in epithelial cancers. For example, *TP53* mutations have been reported in oral, bronchial, bladder, and esophageal epithelia [55]. *KRAS* mutations have been frequently observed in histologically normal lung tissue adjacent to lung tumors, as well as normal mucosa adjacent to *KRAS* mutation–positive colorectal cancer [56–61]. Recently, the analysis of somatic mutation by NGS in benign tissue adjacent to tumors revealed that 80% of samples harbored clonal mutations, with increased frequency associated with older age, smoking, and concurrent mutations in DNA repair genes [62].

Aside from preneoplastic fields, the detection of somatic variants in normal solid tissues has historically proved difficult. The main reasons are the generally slower replicative index compared with the hematopoietic compartment, clonally restrictive tissue architecture, difficulty of tissue access, and low frequency of mutation occurrence. Due to these limitations and insufficient technical resolution, there were very few studies prior to the development of NGS that attempted the analysis of somatic mutation in solid normal tissue not associated with cancer. In a pioneer study using SNP arrays and DNA from autopsy tissue, O’Huallachain et al. reported CNV in a variety of tissues, including pancreas, kidney, brain, liver, small intestine, ovary, and uterus [63]. Importantly, CNVs were more abundant in dividing tissues and often affected genes involved in cell regulation. A later study using SNP arrays in colon tissue reported abundant chromosomal alterations in colon crypts, which are clonal units, and an increase of these alterations with aging [64]. While not the focus of this review, it is noteworthy to mention that clonal expansions in normal aging colon have also been extensively demonstrated by the analysis of mutations in mitochondrial DNA [65]; however, the role these mutations play in clonal expansion and oncogenic potential is poorly understood.

With the advent of NGS, it has become increasingly clear that somatic mutations accumulate with aging in normal tissue, even in individuals who are cancer free. Unequivocal evidence was provided by Martincorena et al. [66], who reported extensive clonal patches of somatic mutations in normal skin, containing mutations in genes such as *NOTCH1*, *NOTCH2*, *NOTCH3*, *TP53*, *FAT1*, and *RBM10*, all well-characterized skin cancer drivers [66]. The development of ecNGS methods has further expanded the ability to detect these somatic mutations in normal tissue because the removal of sequencing errors allows the identification of mutations present at very low frequency [67–71]. For instance, Duplex Sequencing [68,72] was able to detect *TP53* mutations at frequencies <0.01% in peritoneal fluid of women without cancer [24]. Notably, these mutations clustered in cancer-associated *TP53* hotspots and typically resulted in loss of protein activity. Moreover, nonsynonymous mutations were enriched, indicating that, much like their counterparts in blood and skin, these mutations were undergoing positive selection [24]. *TP53* mutations were detected in practically all peritoneal fluid samples analyzed (from women with and without cancer), as well as in paired blood, suggesting that clonal expansions driven by cancer genes are a near-universal feature of aging.

Similar to these reports, a recent study found measurable levels of cancer-associated mutations in numerous “driver genes,” including *KRAS*, *PIK3CA*, *PTEN*, *ARID1A*, *TP53*, and several others, in endometriosis, a benign pathology that only rarely progresses to neoplasia [73]. Seventy-nine percent of patients with endometriosis and no histological sign of cancer harbored somatic mutations. The MAF was as high as 40%, indicating that these mutations are highly prevalent and not limited to small subclonal populations of cells [73]. In a separate study, uterine lavage fluid, which contains mostly endometrial cells, showed abundant cancer driver mutations mostly in *PIK3CA*, *KRAS*, and *PTEN* both in women with and without endometrial cancer [74]. These subclonal events had a MAF between 1% and 30% and were associated with age and postmenopausal status, confirming once again the age-related nature of ACE [74].

Recently, a novel sequencing study of human adult stem cells also demonstrated an age-related accumulation of somatic mutations in small intestine, colon, and liver [75]. Interestingly, while these tissues have very different cancer incidences, their mutation rates were very similar. The types of mutations, however, differed by tissue and reflected the mutation spectra of driver genes found in cancers of each given tissue. Similar overlap in the somatic mutation spectra of normal tissue and its associated cancer was reported by Hoang et al., suggesting that the mutational processes operative in aging also underlie the development of cancer in a given tissue [70].

The age-associated rise of cancer-associated mutations has also been reported in human spermatogonia of healthy men. Offspring of older parents are at an increased risk of having genetic disorders caused by mutations in cancer-associated genes, including Apert syndrome (*FGFR2*) [76], achondroplasia and thanatophoric dysplasia (*FGFR3*) [77], and Costello syndrome (*HRAS*) [78,79]. The increase rate has been attributed to a “selfish selection” mechanism whereby spermatogonia harboring these mutations, which affect the growth factor receptor *RAS* proliferative pathway, outcompete wild-type spermatogonia by clonal expansion, leading to a time-dependent increase in mutant sperm [80].

## The rise of cancer-associated mutations with aging

While the analysis of normal tissue is essential to understand the accumulation of somatic mutation with aging, tumor tissue is highly informative as well, given that a clonal tumor carries the somatic load of the original founder cell. Tumor sequencing analyses have reported the presence of hundreds to thousands of mutations shared by most or all tumor cells, and mutation burden correlated with patient age [75,81–83]. Statistical modeling of tumor sequencing data suggests that half or more of somatic mutations in tumors arise before initiation of the tumor [84]. In addition, there appears to be a correlation between cancer incidence and stem cell divisions across a wide variety of cancer types [85,86]. Even with correction for environmental and hereditary cancer predisposition, replication-derived mutations have been proposed to be responsible for up to two-thirds of the mutations in human cancers [85].

“Mutation signature” analysis provides further support for an underlying mutational process that gives rise to age-associated mutation [87]. Studies using tumor sequencing data and tissue culture of adult stem cells have inferred that tissues generally accumulate approximately 40 mutations/genome/year, independent of tissue of origin, and exhibit two main age-associated mutational signatures [75,83]. The primary signature, composed of C→T/G→A single base substitutions at CpG sites, is consistent with spontaneous deamination of methylated cytosine residues, with the intensity of this signature differing between tissue types and correlating with the rate of replicative turnover [75,83]. The second age-associated mutational signature consists primarily of a mixture of T→C/A→G and C→T/G→A transitions across all sequence contexts. This molecular signature is prevalent only in certain tissue types, and the underlying molecular process is unknown. Importantly, the mutation rates of both signatures do not correlate, indicating that they are likely to have different origins. Nevertheless, given the fact that most cancers show age-dependent associations with one or both of the signatures, they are proposed to represent the accumulation of somatic mutation with aging in human tissues [83]. Reconstruction of cellular lineages during embryogenesis also indicates almost identical mutational signatures, suggesting that the mutagenic processes that give rise to somatic mosaicism begin to operate immediately upon fertilization [88]. However, mouse tissues are reported to have different mutation signatures and rates, indicating that the mutagenic processes that give rise to age-associated mutations may be species specific [1,89]. It is tempting to speculate that these different mutagenic processes may, in part, account for the difference in risk for different cancer types between mice and humans.

Together, these studies have begun to shed light on how cancer-associated mutations arise in normal tissue. A general model can be formed wherein cells mostly accumulate mutations at replication during development and tissue maintenance through life. Although the mutations that accumulate are, by and large, not lethal, they likely influence replicative fitness. In support of this idea, work in yeast indicates that 10% to 20% of diploid strains harboring a heterozygous deletion of just one of the 6,200 genes exhibit a growth disadvantage during competition assays [90]. In the presence of extensive DNA damage and certain oncogenic mutations, cells might activate tumor suppression mechanisms that lead to senescence, further diminishing tissue homeostasis with age. Senescence reduces the number of stem cell lineages, requiring the remaining lineages to divide more frequently to maintain the tissue, thus providing an opportunity to further increase the somatic mutation burden in those cells. The acquisition of a mutation in one of the approximately 140 identified tumor suppressor or oncogenes might lead to a replicative advantage and the expansion of the founder cell into a clone [91]. While these clones appear to be a feature of normal aging, over time they might accumulate additional genetic advantages and develop into cancer. It has been estimated that a replicative advantage of only 0.4% over the course of 20 years could be enough to lead to the acquisition of a tumor [92].

## Concluding remarks

It is estimated that cells in highly proliferative tissues likely contain tens of thousands of mutations by the age of 60, each of which is potentially subjected to selective forces [93]. However, the detection and characterization of these mutations and their functional consequences has been extremely challenging due to their low abundance. Until very recently, only large chromosomal alterations or mutations expanded to at least 10% of the sample could be detected. With the advent of various NGS technologies and methods, the limit of detection has decreased to <0.01%. This resolution has revealed that clonal expansions of cancer-associated mutations is an extremely common, if not universal, condition in somatic tissues. These findings illustrate an ongoing intra-organism process of mutation, selection, and clonal expansion analogous to the inter-organism process that fosters the evolution of species. While this process might culminate in the development of malignancy, it usually does not. However, increased proliferation resulting from a cancer-associated mutation affords more opportunity for additional mutations to occur. Such secondary mutations could act as an accelerant for carcinogenesis by providing sufficient additional clonal diversity through a mutator phenotype or other required driver mutations to overcome intrinsic barriers preventing tumorigenesis. Future studies with larger numbers of individuals of different ages and diversity of normal tissues are essential to elucidate the intricate relationship between the occurrence of somatic mosaicism in aging and cancer.
